# Comparative chloroplast genomes of *Paris* Sect. *Marmorata*: insights into repeat regions and evolutionary implications

**DOI:** 10.1186/s12864-018-5281-x

**Published:** 2018-12-31

**Authors:** Xiaoyang Gao, Xuan Zhang, Honghu Meng, Jing Li, Di Zhang, Changning Liu

**Affiliations:** 1 CAS Key Laboratory of Tropical Plant Resources and Sustainable Use, Xishuangbanna Tropical Botanical Garden, Chinese Academy of Science, Menglun, 666303 Yunnan China; 2 Center for Integrative Conservation, Xishuangbanna Tropical Botanical Garden, Chinese Academy of Sciences, Kunming, 650223 Yunnan China; 3 University of Chinese Academy of Sciences, Beijing, 100049 China

**Keywords:** *Paris* Sect. *Marmorata*, Chloroplast genome, Repeat sequence, Codon usage, Evolutionary rates

## Abstract

**Background:**

Species of *Paris* Sect. *Marmorata* are valuable medicinal plants to synthesize steroidal saponins with effective pharmacological therapy. However, the wild resources of the species are threatened by plundering exploitation before the molecular genetics studies uncover the genomes and evolutionary significance. Thus, the availability of complete chloroplast genome sequences of Sect. *Marmorata* is necessary and crucial to the understanding the plastome evolution of this section and facilitating future population genetics studies. Here, we determined chloroplast genomes of Sect. *Marmorata*, and conducted the whole chloroplast genome comparison.

**Results:**

This study presented detailed sequences and structural variations of chloroplast genomes of Sect. *Marmorata*. Over 40 large repeats and approximately 130 simple sequence repeats as well as a group of genomic hotspots were detected. Inverted repeat contraction of this section was inferred via comparing the chloroplast genomes with the one of *P. verticillata*. Additionally, almost all the plastid protein coding genes were found to prefer ending with A/U. Mutation bias and selection pressure predominately shaped the codon bias of most genes. And most of the genes underwent purifying selection, whereas photosynthetic genes experienced a relatively relaxed purifying selection.

**Conclusions:**

Repeat sequences and hotspot regions can be scanned to detect the intraspecific and interspecific variability, and selected to infer the phylogenetic relationships of Sect. *Marmorata* and other species in subgenus *Daiswa*. Mutation and natural selection were the main forces to drive the codon bias pattern of most plastid protein coding genes. Therefore, this study enhances the understanding about evolution of Sect. *Marmorata* from the chloroplast genome, and provide genomic insights into genetic analyses of Sect*. Marmorata*.

**Electronic supplementary material:**

The online version of this article (10.1186/s12864-018-5281-x) contains supplementary material, which is available to authorized users.

## Background

Herbal medicine is currently becoming increasingly popular to be used in complementary and alternative treatments all over the world. Moreover, herbal medicine is still a major source of healthcare, especially in developing countries, which have limited access to modern medical care [1]. However, the wild resources of plant species are threatened by plundering exploitation with the population growth, particularly the increasing demand for herbal medicine with significant economic value.

The species of *Paris* are famous herbal essence for the elements like steroidal saponins with effective pharmacological therapy. Here, we selected the rare species of the genus *Paris* Section *Marmorata* H. Li to explore the chloroplast genome analyses. The Sect. *Marmorata* comprises two species of perennial medicinal herbs, i.e., *P. marmorata* Stearn and *P. luquanensis* H. Li*. P. marmorata* is mainly distributed in Southwest China (i.e., Yunnan, Sichuan, and Tibet), Nepal, and Bhutan, while *P. luquanensis* is mainly distributed throughout Yunnan (i.e., Luquan and Pingbian) and Sichuan (i.e., Huidong, Puge and Yuexi) [2]. Their morphological characteristics are different from other *Paris* members, as these two species have variegated leaves, grow more slowly and thus are shorter than other *Paris* plants. As the other plants of *Paris*, Sect. *Marmorata* species have been used in oriental medicine for a long time. They contain *Rhizoma Paridis* saponins including diosgenyl and pennogenyl saponins as active ingredients, which are typically used in the treatment of tumors, hemostasis, and inflammation [3–5]. Thus, wild resources of *P. marmorata* and *P. luquanensis* are rapidly declining as consequence of their slow growth and low levels of artificial cultivation, but overexploitation for the economic value. What is more serious is that the wild plants are hard to find, but little is known about the sequence diversity and structure divergence of their chloroplast genomes.

Chloroplasts are essential plant organelles that originated from *Cyanobacteria* by endosymbiosis with the precursor of a nucleated ancestral cell more than 1.2 billion years ago [6]. The circular, double stranded chloroplast genome encodes a set of proteins involved in photosynthesis and other biochemical pathways that are important for plant growth and development, even plant evolution, such as biosynthesis of starch, fatty acids, and pigments [7]. Chloroplast genomes of plants are known to be predominantly uniparentally inherited and highly conserved in both gene order and gene contents [8]. They typically have a quadripartite organization, consisting of two IR regions separated by two regions of unique DNA, LSC region and SSC region [9]. Substitution rates of chloroplast genomes are much lower than those of nuclear DNA, which are even more substantially reduced in the IR regions [10]. Low levels of recombination and primarily uniparental inheritance make chloroplast genomes a valuable source of genetic markers for phylogenetic analyses and useful tools for DNA barcoding [11, 12]. High proportion of SSRs has aroused considerable interest due to the ability to generate highly informative DNA markers [13]. In the light of higher levels of allelic variation of SNPs, SSRs make their use as indicators for species identification, hybridization and introgression analyses [13–15], and they have been widely applied to investigating population differentiation and other plant science studies [16–19]. Therefore, genome-wide comparative analysis of SSRs distribution in chloroplast genomes will lay the foundation for further monitoring gene flow, population differentiation and cytoplasmic diversity of *Paris* plants with intricate hybridization.

Previous study was focus on the phylogeny of *Paris* [20], but lacking of detailed information about genetic variation and molecular structural diversity in these species. The structural and nucleotide sequence variations among chloroplast genomes of Sect. *Marmorata* can be exposed by combining the chloroplast genomes of this section aligned to different reference chloroplast genomes (e.g., *P. verticillata* and *P. polyphylla* var*. yunnanensis*). Thus, chloroplast genomes of Sect. *Marmorata* were sequenced using the Illumina sequencing platform. The comparative analyses of chloroplast genomes will contribute to further investigating genetic diversity and evolution of this section to support conservation management strategies, and assist in the exploration and utilization of *Paris* Sect. *Marmorata* in herbal medicine.

Herein, the aims of this study enable us: (1) to examine sequence variations and screen for hotspot regions in Sect. *Marmorata* chloroplast genomes; (2) to characterize global structural patterns of chloroplast genomes of Sect. *Marmorata*; and (3) to explore codon usage patterns and substitution rates of protein coding genes from chloroplast genomes of Sect. *Marmorata* species.

## Results and discussion

### Chloroplast genome assembly, organization, and features

**Fig. 1:**
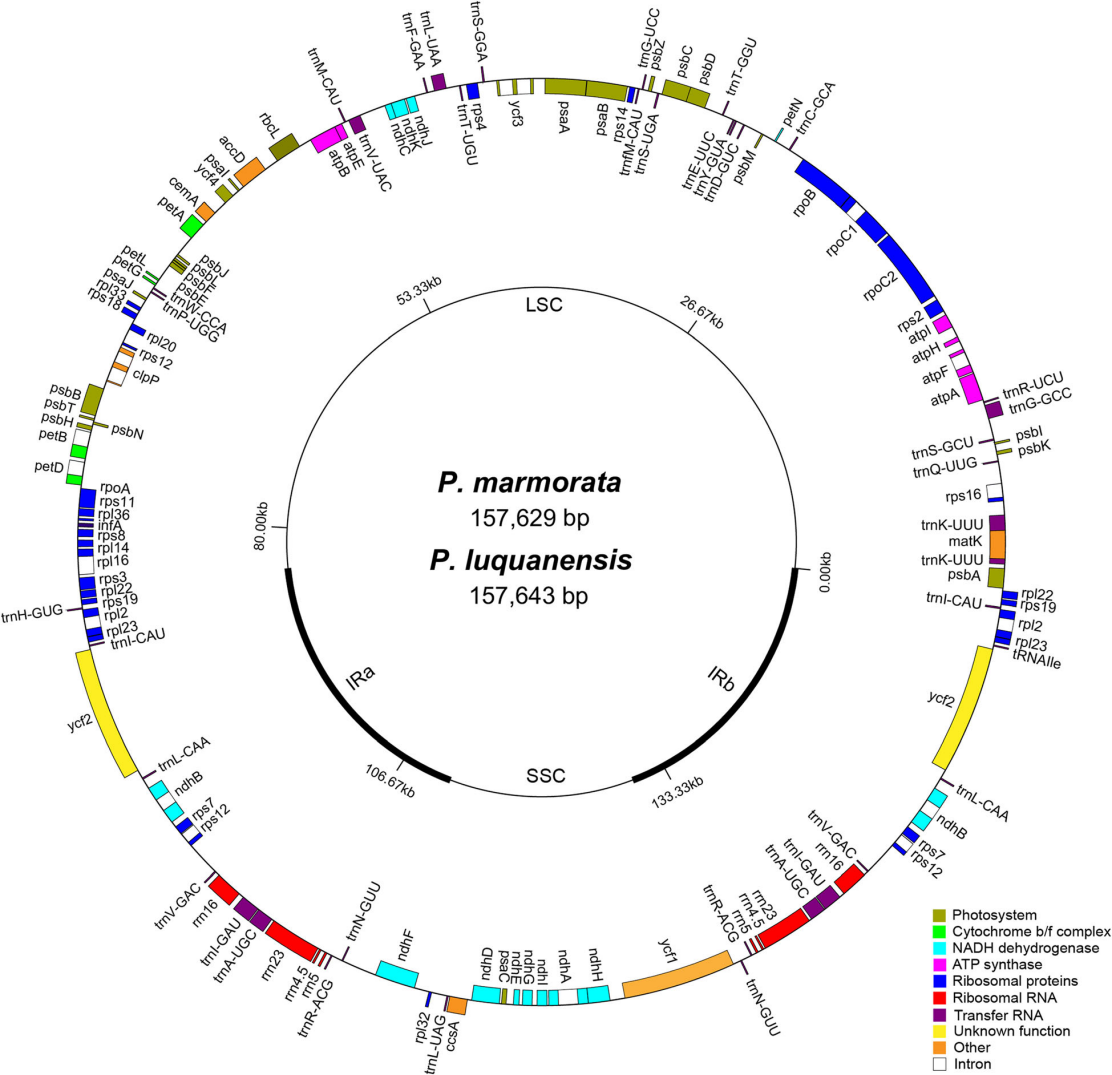
Gene map of chloroplast genomes of *Paris* Sect. *Marmorata*, *P. marmorata* and *P. luquanensis*. Genes belonging to different functional categories are color-coded. Genes shown the inside circle are transcribed clockwise and those located on the outside are transcribed counterclockwise

Illumina paired-end sequencing produced 692 Mb and 762 Mb of data from chloroplast genomes of *P. marmorata and P. luquanensis*, respectively. *P. marmorata*, 1,024,696 reads of a total of 1.54 million were mapped to the reference chloroplast genome with a mean depth of 1,028×. And *P. luquanensis*, 1,662,396 reads of a total of 1.68 million were mapped to the reference chloroplast genome with a mean depth of 1,120×. The N50 contigs length for *P. marmorata* and *P. luquanensis* were 6,990 bp and 36,582 bp, and the complete chloroplast genome sequences were 157,629 bp and 157,643 bp in length, respectively. As the other angiosperms, chloroplast genomes of Sect. *Marmorata* had a typical quadripartite structure consisting of a pair of identical IRs (27,521–27,611 bp), separated by LSC (84,028–84,059 bp) and SSC (18,393–18,529 bp) regions (Fig. 1; Additional file 1: Table S1).

The chloroplast genomes of Sect. *Marmorata* encoded an identical set of 133 predicted functional genes, 113 of which were unique, and 20 were duplicated and located in the IR regions. The 113 unique genes were comprised of 30 tRNA genes, 4 rRNA genes, and 79 protein-coding genes, respectively (Additional file 1: Table S1). Fifteen distinctive genes, including *aptF*, *ndhA*, *petB*, *rpl2*, and *trnA*-*UGC*, contained the single introns; while the genes *clpP*, *rps12*, and *ycf3* contained two introns (Additional file 1: Table S2). These introns share the same splicing mechanism as group II introns [21]. However, different assembly strategies and different reference chloroplast genomes (i.e., *P. verticillata* versus *P. polyphylla* var. *yunnanensis*) led to some differences in gene contents. In the light of sequence similarity, the chloroplast genome of *P. polyphylla* var. *yunnanensis* was regarded as the most similar genome for Sect. *Marmorata* via BLAST in NCBI. In addition, Sect. *Marmorata* species and *P. polyphylla* var. *yunnanensis* are closely related, and they belong to the subgenus *Daiswa*. Notably, comparison of gene contents revealed that two genes were unique to chloroplast genomes sequenced in this study, and another four genes were unique to chloroplast genomes sequenced by Huang et al, respectively (Additional file 2: Figure S1).

A total of 59.41–60.03% of Sect. *Marmorata* chloroplast genomes were protein-coding regions. Overall, 1.83%, 5.74%, and 52.02–52.73% of the genome sequences encoded tRNAs, rRNAs, and proteins, respectively. The remaining sequences consisted of noncoding regions filled with introns, intergenic spacers, and a pseudogene. The *ycf1*-like (*ycf1*^Ψ^) gene in the IRb/SSC junction was found to be the only pseudogene, resulting from an incomplete duplication of the normal functional copy of *ycf1* in the IRa/SSC junction. Similar to the chloroplast genomes of *P. verticillata* and *P. polyphylla* var. *yunnanensis*, the chloroplast genomes of Sect. *Marmorata* were AT-rich with an overall AT content of 62.6%. AT content of LSC (64.25–64.28%), SSC (68.70–70.10%), and IR (58.08–61.10%) regions varied slightly. AT content of genomic regions is probably associated with dynamics of repeat sequences and codon bias of chloroplast protein-coding genes [22, 23]. In general, the chloroplast genome features of Sect. *Marmorata* were similar in the terms of gene content, gene order, introns, intergenic spacers, and AT content.

### Repeat sequence analyses

Repeat regions play an important role in recombination and genomic rearrangements [24, 25]. Eleven sets of repeats were identified in chloroplast genome sequences of *P. marmorata* and *P. luquanensis* using TRF with a 100% match criterion in repeat copies. With a > 90% match criterion, another 10 sets of repeats were identified, yielding 21 total sets detected *in P. marmorata*, with 12 in CDS regions, 8 in intergenic regions and 1 in a span spacer of a CDS region. Similarly, with a > 90% match criterion, another 8 sets of repeats, yielding 19 total sets were detected in *P. luquanensis*, with 9 in CDS regions, 9 in intergenic regions, and 1 in a span spacer of tRNA. The repeats were scattered around the LSC (6–7), SSC (6–8), and IRs (6–7) regions, and they mainly located in the intergenic regions and protein coding regions, including *accD*, *rbcL*, *ycf1* and *ycf2* (Additional file 1: Table S3).

Meanwhile, a total of 30 repeats were identified in chloroplast genome sequences of both *P. marmorata* and *P. luquanensis*, and the sizes of repeats ranged from 63 to 139 bp. Those repeats were scattered around SSC and IRs regions, and they mainly located in the intergenic regions, protein coding regions (*ycf1* and *ycf2*) and *ycf1*^Ψ^ (Additional file 1: Table S4). There were 14 and 21 repeats with 0 hamming distance in *P. marmorata* and *P. luquanensis*, respectively, that is, these were repeats with 100% identity. The output of REPuter was compared with the one of TRF, and the tandem repeats and dispersed repeats (i.e., forward and palindromic) were separately analyzed. The total numbers of those repeats were 51 and 49 for *P. marmorata* and *P. luquanensis*, respectively, in which their copy numbers ranged from 2 to 16. Among the coding regions, the richest in repeats was the *ycf1* gene, which contained 31 and 30 repeats in *P. marmorata* and *P. luquanensis*, respectively. As reported in the other chloroplast genomes, *ycf2* was also rich in repeats, carrying 4 to 5 repeats. These two protein-coding genes and divergent regions are demonstrated to be often associated with repeat events [26]. The above-mentioned repeats can provide valuable information on developing markers for phylogenetic research and population studies.

**Fig. 2:**
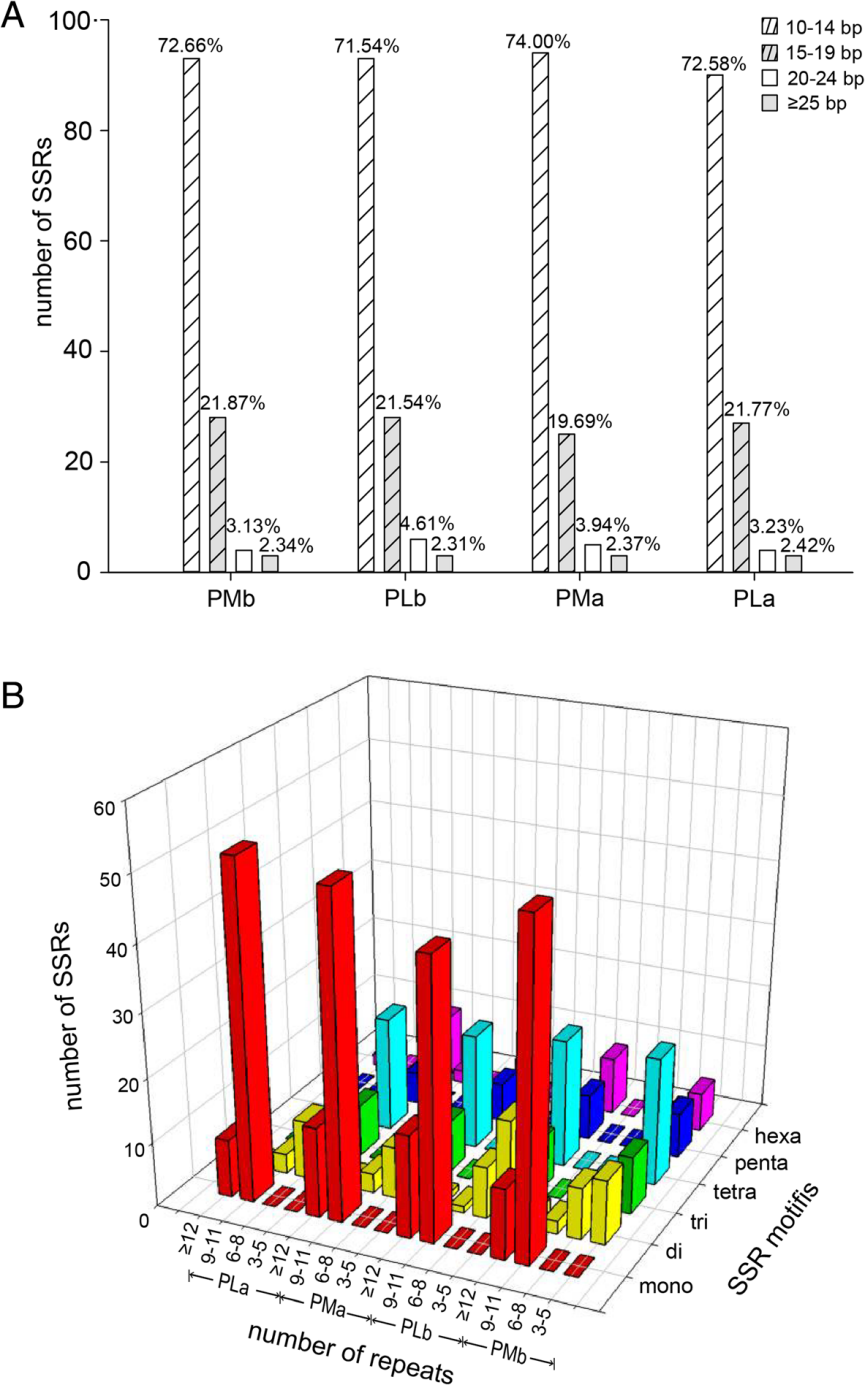
Distribution of microsatellites in chloroplast genomes of Sect. *Marmorata*. **a** length distribution of SSRs in four chloroplast genomes of Sect. *Marmorata*; **b** distribution of chloroplast SSRs repeat number with respect to motif type

SSRs are stretches of small repeating units of DNA occurring in both coding and non-coding regions. SSRs have been used as DNA markers for population genetic studies, due to their polymorphic nature and co-dominant expression [27]. Chloroplast SSRs have been commonly used to characterize genetic variation among plant genotypes [28–31]. In chloroplast genomes of Sect*. Marmorata*, 128–130 SSRs were identified in silico, of which, 59–62 were mononucleotides, 22–26 were dinucleotides, 8–9 were trinucleotides, 20 were tetranucleotides, 7 were pentanucleotides, and 8–10 were hexanucleotides (Additional file 1: Table S5). A total of 22 and 21 SSRs for *P. marmorata* and *P. luquanensis*, respectively, occurred in compound formations that comprised several combinations of SSRs separated by the maximum distance of 100 bp. The most abundant motifs were mononucleotide A/T repeats (contributed to 96.60% of mon-repeat SSRs), comprising about 43.85–46.88% of total repeats. The distribution pattern of six SSR motifs in Sect. *Marmorata* was consistent with that of other *Paris* species (Additional file 2: Figure S2). Gene regions harbored more SSRs (over 40 SSRs) than gene spacer did, and protein coding genes including *rps12*, *ycf1*, etc., harbored more than two SSRs. The length of most SSRs (over 90%) ranged from 10 bp to 19 bp (Fig. 2a; Additional file 3: Table S6). Repeat times of mononucleotide repeats were mostly over 9, and repeat times of most rest five motifs were less than 9 (Fig. 2b). Chloroplast genomes contain conserved genes, but the number of SSRs that they harbor varied. Compared to IR regions, the LSC and SSC regions contain more SSRs, and this finding is consistent with analyses of other angiosperm chloroplast genomes [32–34]. In order to identify whether they are efficient markers for delineating Sect. *Marmorata*, those chloroplastic microsatellites were assessed in silico by calculating Shannon-Winener index and PIC. SSRs in gene regions, such as *ycf1*, etc.; and SSRs in gene spacers such *matK-rps16*, etc., showed high polymorphism (PIC> 0.5), which can be exploited for genetic diversity studies of Sect. *Marmorata* (Additional file 3: Table S7). Moreover, the inferred phylogenetic trees revealed that Sect. *Marmorata* had a well-supported consistent topology, in the light of phylogenetic analyses of some genes and spacers harbored repeat sequences. The regions like *ycf1*-*ndhF* etc., can be the source of genetic markers, assisting in phylogeny and population studies of Sect. *Marmorata*. However, the other sections of the subgenus *Daiswa* showed unstable topologies and the relationships among them were still not very clear, due to lacking of enough representatives of *Paris* (Additional file 2: Figure S3).

### Chloroplast genome comparison of sect. *Marmorata*

**Fig. 3:**
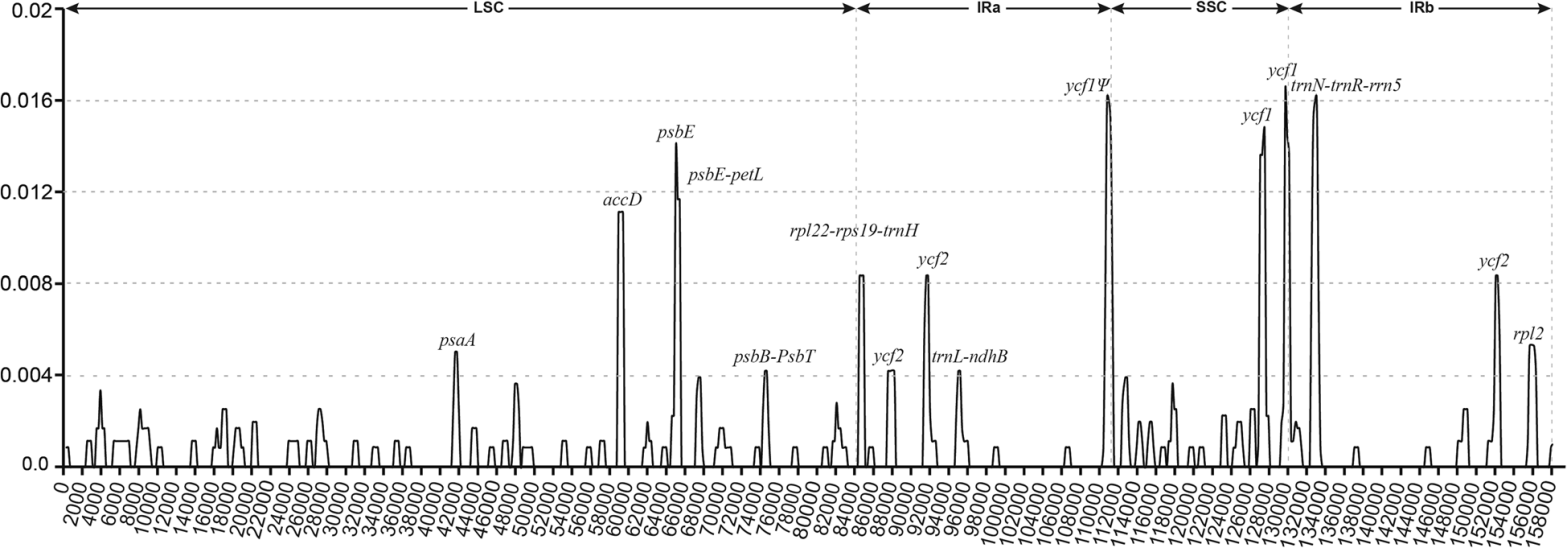
Sliding window analysis of the whole chloroplast genome of Sect. *Marmorata*. X-axis, position of midpoint of a window; Y-axis, nucleotide diversity of each window

The availability of complete chloroplast genomes from the genus *Paris* provides an opportunity to elucidate the chloroplast genome organization and sequence variation of Sect. *Marmorata*. Sequence divergence analyses of four chloroplast genomes of Sect. *Marmorata* revealed *π* values in the range from 0 to 0.01639 with an average of 0.0009, indicating there are a few differences among the four chloroplast genomes (Fig. 3). However, eight protein coding regions (including *psaA*, *accD*, *psbE*, *psbB*, *rps19*, *ycf1*, *ycf2*, and *rpl2*), and seven intergenic regions (including *psbE*-*petL*, *psbB*-*psbT*, *rpl22*-*rps19*, *rps19*-*trnH*-*GUG*, *trnL*-*ndhB*, *trnN*-*GUU*-*trnR*-*ACG*, and *trnR*-*rrn5*) as well as the pseudogene *ycf1*^Ψ^ had higher divergence values (*π* > 0.004), indicating that they harbored more variations than other regions. This is in accordance with the previous study, as a lot of SNPs were detected in the above regions of other *Paris* chloroplast genomes [20]. These regions with highly diverse loci are not random, but they are instead clustered in “hot spots” [35, 36].

**Fig. 4:**
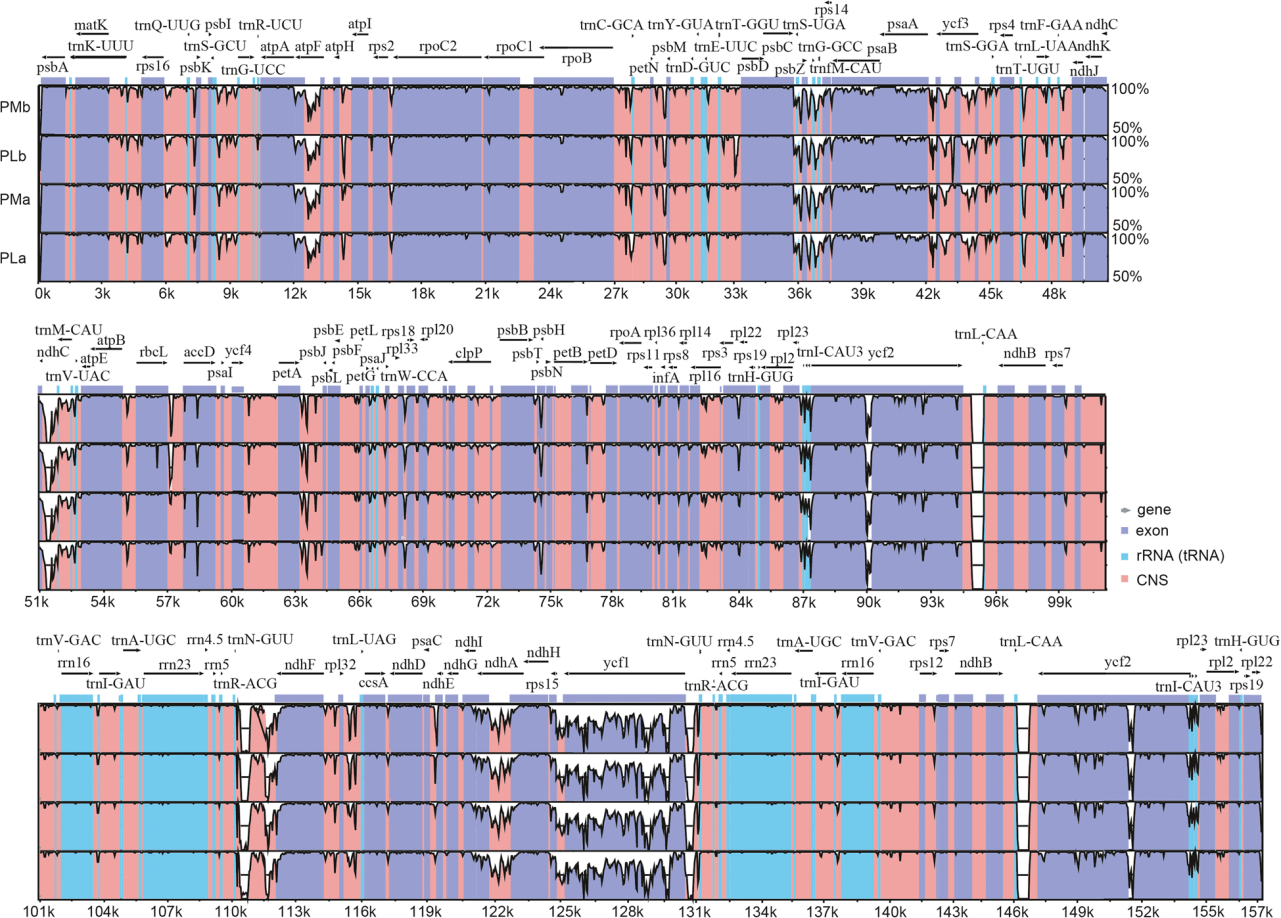
Percent identity plot comparing the chloroplast genomes of Sect. *Marmorata*, with *P. verticillata* as the reference. Vertical scale indicates the percentage of identity, ranging from 50 to 100%. Horizontal axis indicates the coordinates within the chloroplast genomes. Arrows indicate the annotated genes and their transcriptional direction. Genome regions are color coded as exon, rRNA/tRNA, and CNS

The overall sequence identities of four chloroplast genomes of Sect. *Marmorata* were plotted using mVISTA and the chloroplast genome of *P. verticillata* was used as a reference (Fig. 4). The IR regions showed lower sequence divergence than the LSC and SSC regions, possibly due to copy number differences in IR sequences caused by gene conversion [37]. As expected, non-coding regions exhibited higher sequence divergence than coding counterparts, and the most divergent non-coding regions in the chloroplast genomes located in the intergenic spacers. Intergenic regions with high levels of divergence included *atpH/atpI* and *trnH-GUG*/*psbA*. The most divergent coding regions were *ycf1*, *atpF*, and *accD*. Accordingly, universal primers for these intergenic regions could aid in phylogenetic inference and population genetic analysis of Sect. *Marmorata*.

**Fig. 5:**
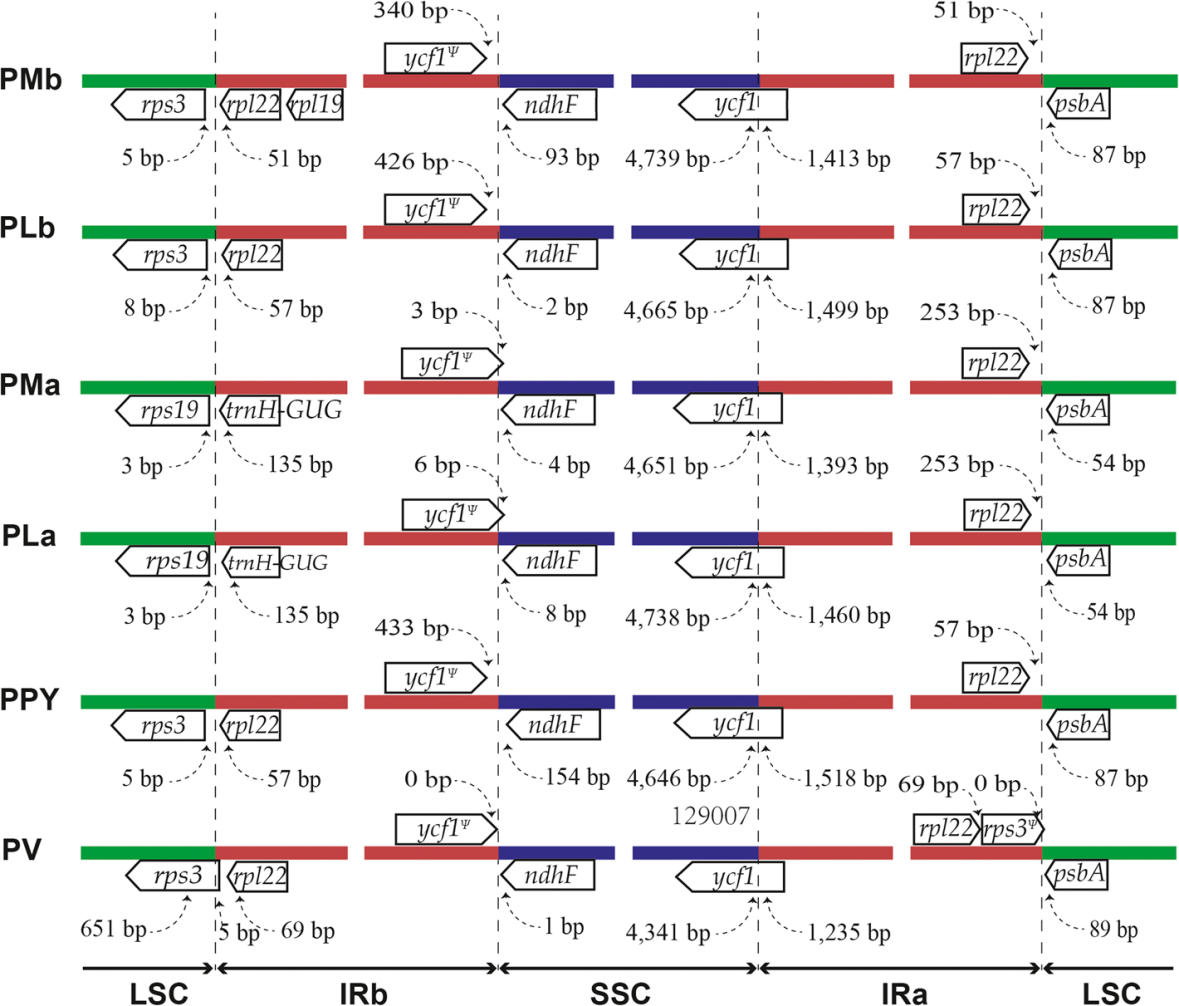
Comparison of boundaries among LSC, SSC and IR regions of chloroplast genomes of Sect. *Marmorata*, *P. verticillata* (PV) and *P. polyphylla* var. *yunnanensis* (PPY). Genes above lines are transcribed forward and those below the lines are transcribed reversely. Ψ indicates a pseudogene. This figure is not to scale

Expansion and contraction of IR regions are common in the evolutionary history of land plants. Indeed, the junctions of LSC/IR and IR/SSC are sometimes regarded as an index of chloroplast genome evolution [38]. To evaluate the potential impact of the junction changes in the chloroplast genomes of Sect. *Marmorata*, we compared the IR boundaries of Sect. *Marmorata* species with that of their references (Fig. 5). In those analyzed chloroplast genomes, IR/SSC boundary was located within the *ycf1* gene, resulting in the formation of *ycf1* pseudogene. *ycf1* observed in subgenus *Daiswa* (Sect. *Marmorata* species and *P. polyphylla* var. *yunnanensis*) was longer than its homologue in the subgenus *Paris* (*P. verticillata*). Additionally, the LSC/IR junction was detected a contraction event in subgenus *Daiswa* (expect for chloroplast genomes of *P. marmorata* and *P. luquanensis* sequenced by Huang et al., namely PMa and PLa)*.* As seen in other chloroplast genomes studies [39], contraction of IR regions in Sect. *Marmorata* has led to changes in the chloroplast genome structure, causing the formation of pseudogenes. It is worth noting that obvious differences in the LSC/IR boundary could also be observed among Sect. *Marmorata* species, because of the different reference chloroplast genomes and assembly methods adopted in the two studies. LSC/IR boundaries of PMa and PLa were next to *rps19* and they were found to be more similar to that of *Veratrum patulum* in the family Melanthiaceae [40]. More sequences are required to further analyze and confirm the IR boundaries of chloroplast genomes of the section. The above analyses also implied that length differences in noncoding regions could affect genome size variation among species of Sect*. Marmorata*.

### Codon usage pattern

Most protein-coding genes employed the standard initiator codon AUG; however, six unusual start codons were also identified, such as TTG (*cemA*) and GTG (*rps19*). Similar noncanonical start codons have been detected in other angiosperms and tree fern plants [41–43]. Furthermore, the codon usage patterns were determined for 71 distinct protein-coding genes in Sect. *Marmorata* chloroplast genomes. Codons of chloroplast genes of Sect. *Marmorata* with A/U at the third position nucleotide were used more frequently than those ending with G/C, according to RSCU values (with a threshold of RSCU > 1). As observed in chloroplast genomes of most land plants, codon usage patterns of this section are likely driven by the composition bias towards the high A/T content.

**Fig. 6:**
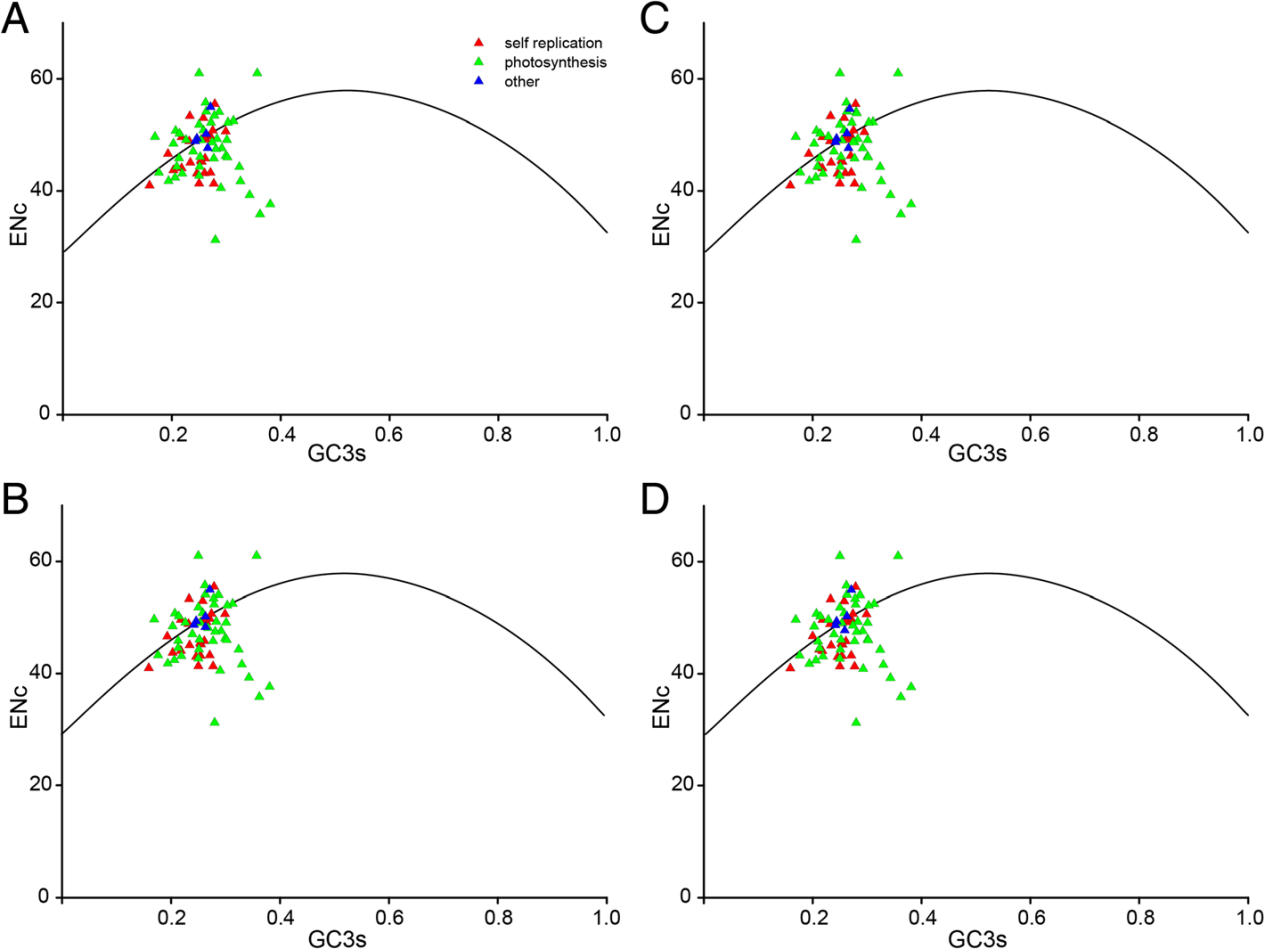
ENc plotted against GC3s. The solid line represents the expected curve of positions of genes when the codon usage was only determined by the GC3s composition. ENc and GC3s plots for Sect. *Marmorata*: **a** PMa, **b** PMb, **c** PLa, **d** PLb

The ENc plots are useful indicators for the factors affecting codon bias. To determine the relative importance of mutation and selection in producing codon usage patterns, ENc values were estimated and plotted against the GC3s values (Fig. 6). The protein-coding genes from four chloroplast genomes of Sect. *Marmorata* species shared the analogous codon bias patterns. A small number of genes from three functional categories exactly followed the standard curve, which certainly originated from extreme GC compositional constraints, and their codon biases were consistent with by mutation bias [44]. In particular, more than half of genes were below the curve, suggesting that the selection forces predominantly influenced these genes, and that natural selection had more influence on codon bias of chloroplast genome [45]. Intriguingly, genes associated with photosynthesis showed a discrete distribution, which implied that other factors such as gene length and gene expression level also influenced the codon bias.

### Evolutionary rates of sect. *Marmorata* chloroplast genes

**Fig. 7:**
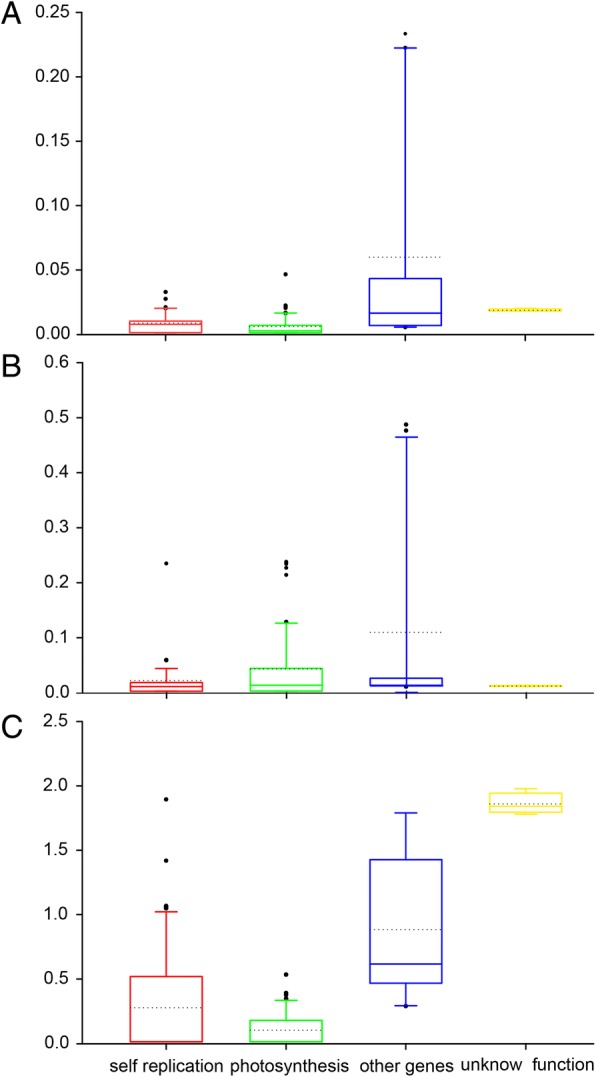
Comparison of nonsynonymous and synonymous substitution rates and their ratios of protein-coding genes. **a**-**c** denote *K*_a_, *K*_s_, and *K*_a_/*K*_s_, respectively. Red highlight boxplots indicate genes involved in self-replication, green ones indicate photosynthesis genes, and blue ones indicate other genes

*K*_a_, *K*_s_ and *K*_a_/*K*_s_ are valuable indicators of evolutionary rates and natural selection [46]. To better understand the evolutionary forces shaping chloroplast genes of Sect. *Marmorata*, the substitution rates (*K*_a_ and *K*_s_, and *K*_a_*/K*_s_ ratios) of 77 conserved protein-coding genes were estimated using *P. verticillata* as reference (Additional file 2: Figure S4). Overall, chloroplast protein-coding genes from different Sect. *Marmorata* species had similar substitution rates. However, significant high *K*_a_*/K*_s_ ratios of gene *psbB*, *rpl20* and *rps2* from *P. luquanensis* sequenced simultaneously (PLa) were discovered, which were obviously different from those values of P*. luquanensis* sequenced in this study (PLb), and *P. marmorata* sequenced simultaneously (PMa) and *P. marmorata* sequenced in this study (PMb). And the *K*_a_*/K*_s_ values of most protein-coding genes were less than 1, except for *rpoC1*, *rps11*, *accD*, *clpP*, and *ycf2*, which indicated that most of the genes experienced a purifying selection. The *K*_a_*/K*_s_ ratios of protein-coding genes in the IR regions were higher than those in LSC and SSC regions. Additionally, a set of genes with *K*_a_*/K*_s_ values exceeding 45 or *K*_a_*/K*_s_ values that were otherwise incalculable (NA), included *rps7*, *rps19*, and *rps12* in the IR region (with *K*_a_*/K*_s_ values exceeding 50), *aptI*, *psaI*, *psbF*, *psbN*, *rpl16*, *rpl20*, *rps18*, *rps2*, and *rps4* in LSC region (with *K*_a_*/K*_s_ values exceeding 45), and *petL*, *petN*, *ndhJ*, *psaJ*, *psbI*, *psbJ*, *psbL*, *rpl33*, *rpl36*, *rps16*, *ndhE*, and *rpl32* (*Ka/Ks* values were “NA”). High and incalculable values occurred when the *K*_s_ values were extremely low or when there were no substitutions in the alignment (i.e., there was a 100% match), respectively. For both cases, the values of NA and ratios over 45 were manually changed to 0 [47]. After sorting the genes into functional categories and groups, widely variable substitution rates among 77 genes were discovered (Fig. 7; Additional file 2: Figure S5). *K*_a_ and *K*_a_/*K*_s_ of genes related to photosynthesis were apparently lower than that of genes related to self-replication as well as other types of genes. It can speculate that genes involved in photosynthesis tend to be more divergent than genes related to self-replication and other genes, and photosynthetic genes are under somewhat relaxed purifying selection [47]. Although the causes and consequences of evolutionary rate differences among protein-coding genes remain a subject of debate, disparities in generation time, relaxed selection, length of encode protein products, gene expression level, and gene function have all been suggested as potential explanations of such differences [48–51].

## Conclusions

Complete chloroplast genome sequences of Sect. *Marmorata*, i.e., *P. marmorata* and *P. luquanensis* were assembled, annotated and explored subsequent genome-wide comparative analyses. The chloroplast genomes exhibit a typical quadripartite structure of LSC and SSC regions separated by a pair of IRs, and they share similar features in the terms of gene organization and AT-rich content. Large repeats, polymorphic SSR loci, as well as genes and intergenic regions with high levels of variability were determined. Those repeat motifs and hotspot regions can be selected to study both intraspecific and interspecific variability, and they also can aid in inferring phylogenetic relationships of Sect. *Marmorata* and other species of subgenus *Daiswa*. Non-coding regions like some intergenic spacers exhibited significantly higher sequence divergence than most coding regions, and the divergent lengths of noncoding regions affected chloroplast genome size variation among species. Almost all the chloroplastic protein-coding genes bias ended with A/U. Mutations and selection forces, particularly natural selection, shaped the codon bias pattern of most genes. Most of these mentioned genes were predicted to have a signature of purifying selection, whereas photosynthetic genes experienced a relatively relaxed purifying selection, whose codon bias and evolutionary rate were also driven by other factors such as gene expression level and gene length. Additionally, chloroplast genomes sequenced in this study and those simultaneously sequenced did have differences in both gene content and structure, which brought different results of quadripartite boundaries, substitution rates, and selective pressure. Nevertheless, chloroplast genomes sequenced together with the ones sequenced simultaneously can provide insights into both the genetic relationships among Sect. *Marmorata* and the other species of *Paris*. Moreover, the results aid to expand the current understanding of the evolutionary history of *Paris*, particularly Sect. *Marmorata*.

## Methods

### Taxon sampling, DNA extraction and sequencing

*P. marmorata* and *P. luquanensis* analyzed in this study were cultivated and collected in green house (Kunming) of Xishuangbanna Tropical Botanical Garden. No specific permits are required for sampling. Total genomic DNA was extracted from 100 mg of fresh healthy leaves using a modified CTAB method [52, 53], and the quality of each sample was assessed by agarose gel electrophoresis. The whole chloroplast genomes were amplified using long-range PCR and nine universal primer pairs according to the procedure outlined by Yang et al [53]. Then, six μg of purified PCR products was mixed and fragmented to construct short insert libraries (measuring 200–500 bp in length) according to the procedures outlined in the Illumina manual. The paired-end libraries were then sequenced using Illumina MiSeq 2000 platform (Illumina, San Diego, CA, USA) at Germplasm Bank of Wild Species, Kunming Institute of Botany, Chinese Academy of Sciences.

### Assembly and annotation

Raw reads were filtered with the quality control program NGSQCToolkit v2.3.3 to obtain high quality Illumina data (the cut-off value for percentage of read length was 80, and the cut-off value for PHRED quality score was 30) and adaptor-free reads. Filtered reads were then assembled into contigs using SPAdes v3.6.1. Outputted contigs were aligned with the reference *P. polyphylla* var. *yunnanensis* chloroplast genome (Genbank accession No. KT805945) Contigs were then aligned with the reference genome to assemble each chloroplast genome sequence using Geneious v4.8.4. Assembled genome sequences were annotated using the online tool DOGMA and Geneious v4.8.4 [54, 55], and then annotated sequences were manually edited for start and stop codons. All tRNA genes were further confirmed by the online tRNAscan-SE search server [56]. The annotated chloroplast genomes were deposited in GenBank with accession numbers: *P. marmorata* (MF495705) and *P. luquanensis* (MF417768). The annotated GenBank files of the two *Paris* chloroplast genomes were uploaded to obtain gene maps using the online tool GenomeVx [57].

### Repeat sequence identification

Repeat elements in chloroplast genomes of *P. marmorata* and *P. luquanensis* were investigated using three different programs. The position and type of SSR were ascertained using the microsatellite identification tool MISA v1.0 [58], and each repeat sequence length was screened to be ≥10 bp. SSRs were identified with thresholds of 10, 5, 4, 3, 3, and 3 repeat units for mono-, di-, tri-, tetra-, penta-, and hexa-nucleotides, respectively. Diversity of chloroplast SSR markers were further estimated using the Shannon-Winener index and PIC. Tandem repeat sequences (> 10 bp in length) were identified with TRF v4.09 [59], with parameters of 2, 7, and 7 for matches, mismatches and indels, respectively. The minimum alignment score and maximum period size were set to 50 and 500, respectively. Meanwhile, the size and location of both forward and inverted/palindromic repeats were determined using REPuter v1.0 [60]. The parameters were set with a minimal repeat size of 30 bp, hamming distance of 3 kb, and 90% sequence identity threshold. Gene and intergenic spacer regions harbored repeat sequence were extracted, on the basis of the loci of repeats. These regions were then applied to infer phylogenetic relationships with Neighbor-Joining algorithm in MEGA v6.06 and Maximum Likelihood algorithm of RAxML v7.2.6.

### Comparison of chloroplast genome sequences

To investigate the sequence divergence among Sect. *Marmorata* chloroplast genomes, several released chloroplast genomes were retrieved from NCBI: *P. marmorata* (NC_033516, denoted by PMa), *P. luquanensi*s (NC_033514, denoted by PLa), and *P. verticillata* (NC_024560, denoted by PV). Four chloroplast genome sequences of Sect. *Marmorata* were aligned using MAFFT v7.305b [61] and were manually adjusted using Se-Al v2.0 [62]. A sliding window analysis was conducted to compare *π* among the chloroplast genomes of Sect. *Marmorata*, using DnaSP v5.0 [63]. The window length was 600 bp with a 200 bp step size. To reveal both inter- and intra-specific variations, the full alignments of chloroplast genome sequences of Sect. *Marmorata* species and *P. verticillata* were visualized with Shuffle-LAGAN mode in mVISTA program [64].

### Codon usage and substitution rate calculation

RSCU, GC3s, and ENc for 71 protein-coding genes were calculated using CodonW v1.4.4 [65]. Then, the relationships between ENc and GC3s were analyzed. *K*_a_, *K*_s_ and their ratios *K*_a_*/K*_s_ were estimated with ParaAT v2.0 and KaKs_caculator v2.0 [66, 67]. These substitution analyses of 77 conserved protein-coding genes from chloroplast genomes of Sect. *Marmorata* species were implemented, using alignments with *P. verticillata*. Boxplots were constructed for each functional category/group, and plotted with SigmaPlot v13.0.

## Additional files

Additional file 1: **Table S1.** Summary of chloroplast genome characteristics of Sect*. Marmorata.* **Table S2.** List of gene function in the chloroplast genomes*.* **Table S3.** Distribution of tandem repeats from the chloroplast genomes*.* **Table S4.** Distribution of dispersed, forward and inverted/palindromic repeats from chloroplast genomes*.* **Table S5.** Type and number of SSRs in the chloroplast genomes*. (PDF 396 kb)*

Additional file 2: **Figure S1.** Venn diagram analysis for gene composition from chloroplast genomes of Sect. *Marmorata* via different assembly methods and reference genomes. **Figure S2.** Six kinds of SSR motifs in fifteen *Paris* chloroplast genomes. *P. polyphylla* var. *yunnanensis0* was sequenced by Song et al (2015). *P. marmorata0*, *P. luquanensis0*, *P. polyphylla* var. *yunnanensis* were sequenced by Huang et al (2016). *P. marmorata* and *P. luquanensis* were sequenced in this study. **Figure S3.** Phylogenetic trees of genes spacer regions harbored repeat sequence, using NJ (bootstrap values on the left of slashes) and ML (bootstrap values on the right of slashes) algorithms. **Figure S4.** The *K*_a_/*K*_s_ ratios of 71 protein-coding genes from chloroplast genome of Sect. *Marmorata*. Red bars and blue bars denote *P. marmorata* (PMa) and *P*. *luquanensis* (PLa) sequenced previously; green bars and yellow bars denote *P. marmorata* (PMb) and *P*. *luquanensis* (PLb) sequenced in this study. **Figure S5.** Comparison of *K*_a_, *K*_s_, and *K*_a_/*K*_*s*_ ratios of Sect. *Marmorata* chloroplast genes. (A-C) denote *K*_a_, *K*_s_, and *K*_a_/*K*_s_, respectively. Red highlight boxplots indicate photosynthesis genes, green ones indicate genes involved in self-replication, and blue ones indicate other genes. SR: small subunit of ribosome, LR: large subunit of ribosome, DR: DNA-dependent RNA, TF: translational initiation factor, ND: NAPH dehydrogenase, PI: photosystem I, PII: photosystem II, CC: cytochrome b/f complex, AS: ATP synthase gene, LS: large subunit of rubisco, SA: subunit of acetyl-CoA, CS: cytochrome synthesis, CT: c-type cytochrome synthesis, PR: protease, MA: maturase, CO: conserved ORF. (PDF 793 kb)

Additional file 3: **Table S6.** Number of SSRs in fifteen chloroplast genomes of *Paris* species. **Table S7.** Polymorphism of SSRs in chloroplast genomes of Sect. *Marmorata*. (XLSX 17 kb)

## Abbreviations

CDS: Coding DNA sequence
CNS: Conserved noncoding sequence
DOGMA: Dual Organellar GenoMe Annotator
ENc: Effective number of codons
GC3s: GC content on the third synonymous codon position
IR: Inverted repeat
*K*_*a*_: Nonsynonymous substitutions per non-synonymous site
*K*_*s*_: Synonymous substitutions per synonymous site
LSC: Large single copy
PIC: Polymorphic information content
RSCU: Relative synonymous codon usage
SNP: Single nucleotide polymorphism
SSC: Small single copy
SSR: Simple sequence repeat
TRF: Tandem Repeats Finder
*π*: Nucleotide diversity
