# Supplementary material for: Comparative chloroplast genomes of Paris Sect. Marmorata: insights into repeat regions and evolutionary implications
Source: BMC Genomics. 2018 Dec 31;19(Suppl 10):878. doi: 10.1186/s12864-018-5281-x (PMC6311911; doi:10.1186/s12864-018-5281-x)
Supplement: Supplementary file 1 — Table S1. Summary of chloroplast genome characteristics of Sect. Marmorata. Table S2. List of gene function in the chloroplast genomes. Table S3. Distribution of tandem repeats from the chloroplast genomes. Table S4. Distribution of dispersed, forward and inverted/palindromic repeats from chloroplast genomes. Table S5. Type and number of SSRs in the chloroplast genomes. (PDF 396 kb) [file 12864_2018_5281_MOESM1_ESM.pdf]

Table S1. Summary of chloroplast genome characteristics of Sect. *Marmorata*.

**Genome feature**	***P. marmorata***	***P. luquanensis***
Total length (bp)	157,629	157,643
LSC length (bp)	84,059	84,028
SSC length (bp)	18,529	18,393
IR length (bp)	27,521	27,611
Protein-coding genes(bp)	93,958	93,968
Ribosomal RNAs(bp)	9,052	9,050
Transfer RNAs(bp)	10,833	10,817
Introns(bp)	19,878	19,886
Spacers(bp)	23,908	23,922
A+T content (%)		
LSC	64.28	64.25
SSC	68.70	70.10
IR	58.08	61.10
Genome	62.64	62.62
Protein-coding genes	59.93	59.92
Transfer RNA genes	53.28	53.27
Ribosomal RNA genes	45.02	45.02
Total number of genes	133	133
Transfer RNAs	38	38
Ribosomal RNAs	8	8
Protein coding genes	87	87
Genes duplicated in IRa	20	20

Table S2. List of gene function in the chloroplast genomes.

Category	Gene group	Gene name					
Self replication							
	Ribosomal RNA genes (4)	*rrn4.5*[#]	*rrn5*[#]	*rrn 16*[#]	*rrn23*[#]		
		trnA-UGC[#*]	*trnC-GCA*	*trnD-GUC*	*trnE-UUC*	*trnF-GAA*	*trnfM-CAU*
		trnG-GCC[*]	*trnG-UCC*[*]	*trnH-GUG*[#]	*trnI-CAU*[#]	*trnI-GAU*[#*]	*trnK-UUU*[*]
	Transfer RNA genes (30)	*trnL-CAA*[#]	*trnL-UAA*[*]	*trnL-UAG*	*trnM-CAU*	*trnN-GUU*[#]	*trnP-UGG*
		trnQ-UUG	*trnR-ACG*[#]	*trnR-UCU*	*trnS-GCU*	*trnS-GGA*	*trnS-UGA*
		trnT-GGU	*trnT-UGU*	*trnV-GAC*[#]	*trnV-UAC*[*]	*trnW-CCA*	*trnY-GUA*
	Small subunit of ribosome (11)	*rps2*	*rps3*	*rps4*	*rps7*[#]	*rps8*	*rps11*
		rps12[#]	*rps14*	*rps16*	*rps18*	*rps19*[#]	
	Large subunit of ribosome (9)	*rpl2*	*rpl14*	*rpl16*	*rpl20*	*rpl22*[#]	*rpl23*[#]
		rpl32	*rpl33*	*rpl36*			
	DNA-dependent RNA polymerase (4)	*rpoA*	*rpoB*	*rpoC1*	*rpoC2*		
	Translational initiation factor (1)	*infA*					
Photosynthesis							
	NADH dehydrogenase (11)	*ndhA*	*ndhB*[#]	*ndhC*	*ndhD*	*ndhE*	*ndhF*
		ndhG	*ndhH*	*ndhI*	*ndhJ*	*ndhK*	
	Photosystem I (7)	*psaA*	*psaB*	*psaC*	*psaI*	*psaJ*	*ycf3*
		ycf4					
		psbA	*psbB*	*psbC*	*psbD*	*psbE*	*psbF*
	Photosystem II (15)	*psbH*	*psbI*	*psbJ*	*psbK*	*psbL*	*psbM*
		psbN	*psbT*	*psbZ*			
	Cytochrome b/f complex (6)	*petA*	*petB*	*petD*	*petG*	*petL*	*petN*
	ATP synthase (6)	*atpA*	*atpB*	*atpE*	*atpF*	*atpH*	*atpI*
	Large subunit of rubisco (1)	*rbcL*					
Other genes							
	Maturase (1)	*matK*					

	Envelope membrane protein (1)	*cemA*
	Subunit of acetyl-CoA (1)	*accD*
	c-type cytochrome synthesis (1)	*ccsA*
	Component of TIC complex (1)	*ycf1*
	Protease (1)	*clpP*
Unknown function		
	Conserved ORF (2)	*ycf2*[#]

* denotes Genes with introns; # denotes duplicated genes.

Table S3. Distribution of tandem repeats from the chloroplast genomes.

**species**	**region**	**location**	**start**	**end**	**pattern size**	**copy num**	**match percent**
P. marmorata	LSC	spacer	31410	31453	22	2	95
	LSC	spacer	32132	32185	27	2	100
	LSC	spacer	32769	32894	63	2	100
	LSC	rbcL	56647	56742	48	2	97
	LSC	accD	58650	58692	6	7.2	100
	LSC	spacer	61066	61107	21	2	100
	LSC	spacer	68423	68494	34	2.1	100
	IRb	spacer	87134	87249	24	4.8	100
	IRb	ycf1	89815	89919	42	2.5	98
	IRb	ycf1	90042	90138	45	2.2	92
	IRb/SSC	spacer/ndhF	111580	111770	100	1.9	100
	SSC	spacer	119154	119268	58	2	100
	SSC	spacer	125022	125066	19	2.3	92
	SSC	ycf1	126176	126345	57	3	99
	SSC	ycf1	126748	126789	21	2	100
	SSC	ycf1	128766	128887	9	13.6	99
	SSC	ycf1	129463	129620	30	5.1	95
	SSC	ycf1	129700	129740	12	3.4	100
	IRa	ycf2	151550	151646	45	2.2	92
	IRa	ycf2	151769	151873	42	2.5	98
	IRa	ycf2	154439	154554	24	4.8	100
P. luquanensis	LSC	spacer	8364	8416	21	2.5	90
	LSC	spacer	8742	8783	21	2	100
	LSC	spacer	31378	31421	22	2	95
	LSC	accD	58631	58673	6	7.2	100
	LSC	spacer	61047	61088	21	2	100
	LSC	spacer	68404	68475	34	2.1	100
	IRb	spacer	87110	87225	24	4.8	100
	IRb	ycf2	90005	90101	45	2.2	92
	IRb	spacer	109916	110049	70	1.9	100
	SSC	spacer	125002	125064	19	3.3	97
	SSC	ycf1	126174	126343	57	3	96
	SSC	ycf1	126746	126787	21	2	100
	SSC	ycf1	128738	128885	9	16.4	100
	SSC	ycf1	129466	129630	30	5.3	95
	SSC	ycf1	129710	129750	12	3.4	100
	IRa	spacer	129710	131756	70	1.9	100
	IRa	ycf2	151571	151667	45	2.2	92
	IRa	ycf2	151790	151894	42	2.5	98
	IRa	spacer/trnI-CAU	154447	154562	24	4.8	100

Table S4. Distribution of dispersed, forward and inverted/palindromic repeats from chloroplast genomes.

Species	Repeats	LSC	IRb	SSC	IRa
P. marmorata	num	1	10	11	8
	size	63	66-106	68-116	68-126
	location	intergenic spacer$_{(1)}$	intergenic spacer$_{(2)}$<br>ycf1$^{\Psi}_{(8)}$	intergenic spacer$_{(1)}$<br>ycf1$_{(10)}$	ycf1$_{(6)}$<br>ycf2$_{(2)}$
P. luquanensis	num	0	10	12	8
	size	-	64-126	67-139	68-126
	location	-	intergenic spacer$_{(3)}$<br>ycf1$^{\Psi}_{(7)}$	ycf1$_{(12)}$	ycf1$_{(6)}$<br>ycf2$_{(2)}$

$^{\Psi}$ indicates a pseudogene.

Table S5. Type and number of SSRs in the chloroplast genomes.

SSRs	Mono	Di	Tri	Tetra	Penta	Hexa	Total
P. marmorata							
Total Counts	62	22	9	20	7	8	128
Total Repeat Length (bp)	671	286	117	292	115	192	1673
Density (bp/kb)	4.26	1.81	0.74	1.85	0.73	1.22	10.61
Proportion among other SSR (%)	40.11	17.10	6.99	17.45	6.87	11.48	100
Mean Length (bp)	10.82	13.00	13.00	14.60	16.43	24.00	13.07
				AAAG/CTTT		AAAGAG/CTCTTT	
				AAAT/ATTT		AAAGCG/CGCTTT	
				AAGG/CCTT		AAATAT/ATATTT	
		AC/GT	AAG/CTT	AATC/ATTG	AAATG/ATTTC	AAATCC/ATTTGG	
	A/T	AG/CT	AAT/ATT	AATG/ATTC	AATAC/ATTGT	AAGAGG/CCTCTT	
Type of SSR motifs	C/G	AT/AT	ACT/AGT	AGAT/ATCT	AATAT/ATATT	AATGAC/ATTGTC	
P. luquanensis							
Total Counts	59	26	8	20	7	10	130
Total Repeat Length (bp)	651	306	105	292	106	246	1706
Density (bp/kb)	4.13	1.94	0.67	1.85	0.67	1.56	10.82
Proportion among other SSR (%)	38.16	17.94	6.15	17.12	6.21	14.42	100
Mean Length (bp)	11.03	11.77	13.13	14.60	15.14	24.60	13.12
				AAAG/CTTT		AAAGAG/CTCTTT	
				AAAT/ATTT		AAAGCG/CGCTTT	
		AC/GT	AAG/CTT	AAGG/CCTT	AAATG/ATTTC	AAATAT/ATATTT	
	A/T	AG/CT	AAT/ATT	AATG/ATTC	AATAC/ATTGT	AAATCC/ATTTGG	
Type of SSR motifs	C/G	AT/AT	ACT/AGT	AGAT/ATCT	AATAT/ATATT	AAGAGG/CCTCTT	

AATACT/AGTATT
AATGAC/ATTGTC
ACACAT/ATGTGT
