# Supplementary material for: Comparative chloroplast genomes of Paris Sect. Marmorata: insights into repeat regions and evolutionary implications
Source: BMC Genomics. 2018 Dec 31;19(Suppl 10):878. doi: 10.1186/s12864-018-5281-x (PMC6311911; doi:10.1186/s12864-018-5281-x)
Supplement: Supplementary file 2 — Figure S1. Venn diagram analysis for gene composition from chloroplast genomes of Sect. Marmorata via different assembly methods and reference genomes. Figure S2. Six kinds of SSR motifs in fifteen Paris chloroplast genomes. P. polyphylla var. yunnanensis0 was sequenced by Song et al (2015). P. marmorata0, P. luquanensis0, P. polyphylla var. yunnanensis were sequenced by Huang et al (2016). P. marmorata and P. luquanensis were sequenced in this study. Figure S3. Phylogenetic trees of genes spacer regions harbored repeat sequence, using NJ (bootstrap values on the left of slashes) and ML (bootstrap values on the right of slashes) algorithms. Figure S4. The Ka/Ks ratios of 71 protein-coding genes from chloroplast genome of Sect. Marmorata. Red bars and blue bars denote P. marmorata (PMa) and P. luquanensis (PLa) sequenced previously; green bars and yellow bars denote P. marmorata (PMb) and P. luquanensis (PLb) sequenced in this study. Figure S5. Comparison of Ka, Ks, and Ka/Ks ratios of Sect. Marmorata chloroplast genes. (A-C) denote Ka, Ks, and Ka/Ks, respectively. Red highlight boxplots indicate photosynthesis genes, green ones indicate genes involved in self-replication, and blue ones indicate other genes. SR: small subunit of ribosome, LR: large subunit of ribosome, DR: DNA-dependent RNA, TF: translational initiation factor, ND: NAPH dehydrogenase, PI: photosystem I, PII: photosystem II, CC: cytochrome b/f complex, AS: ATP synthase gene, LS: large subunit of rubisco, SA: subunit of acetyl-CoA, CS: cytochrome synthesis, CT: c-type cytochrome synthesis, PR: protease, MA: maturase, CO: conserved ORF. (PDF 793 kb) [file 12864_2018_5281_MOESM2_ESM.pdf]

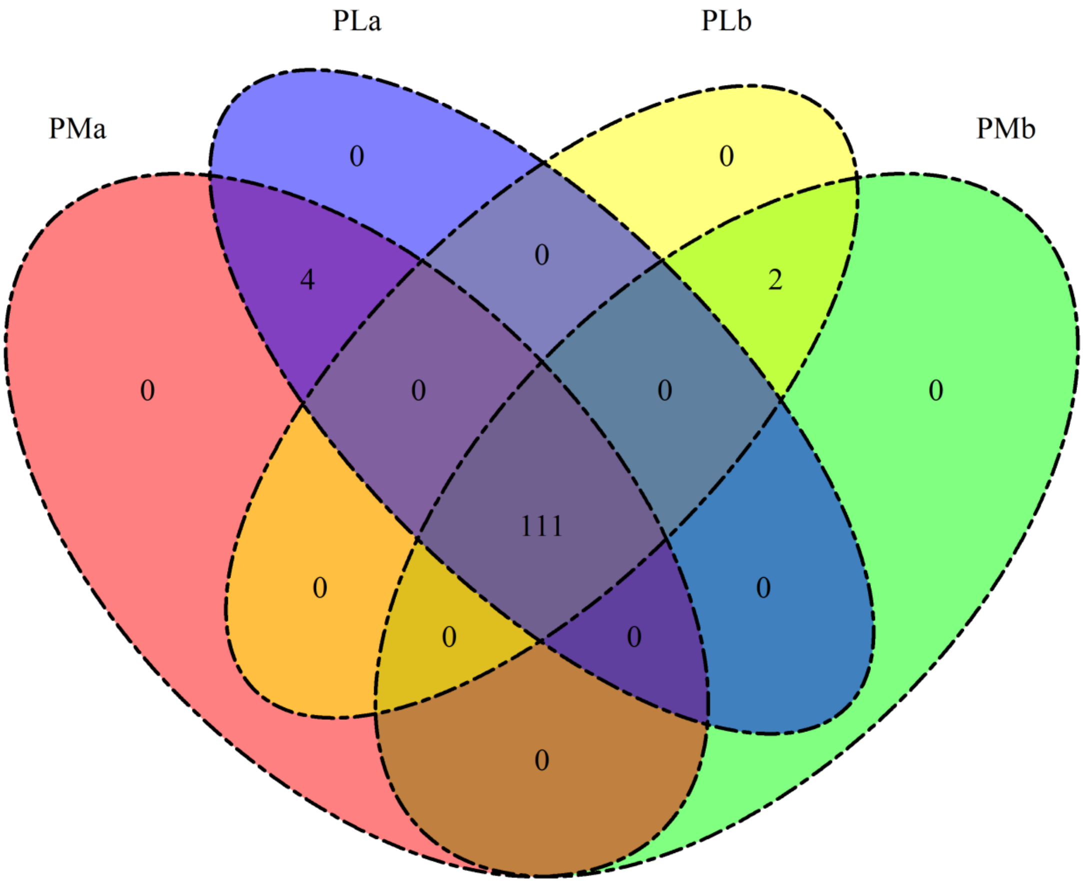

Figure S1 Venn diagram analysis for gene composition from chloroplast genomes of Sect. *Marmorata* via different assembly methods and reference genomes.

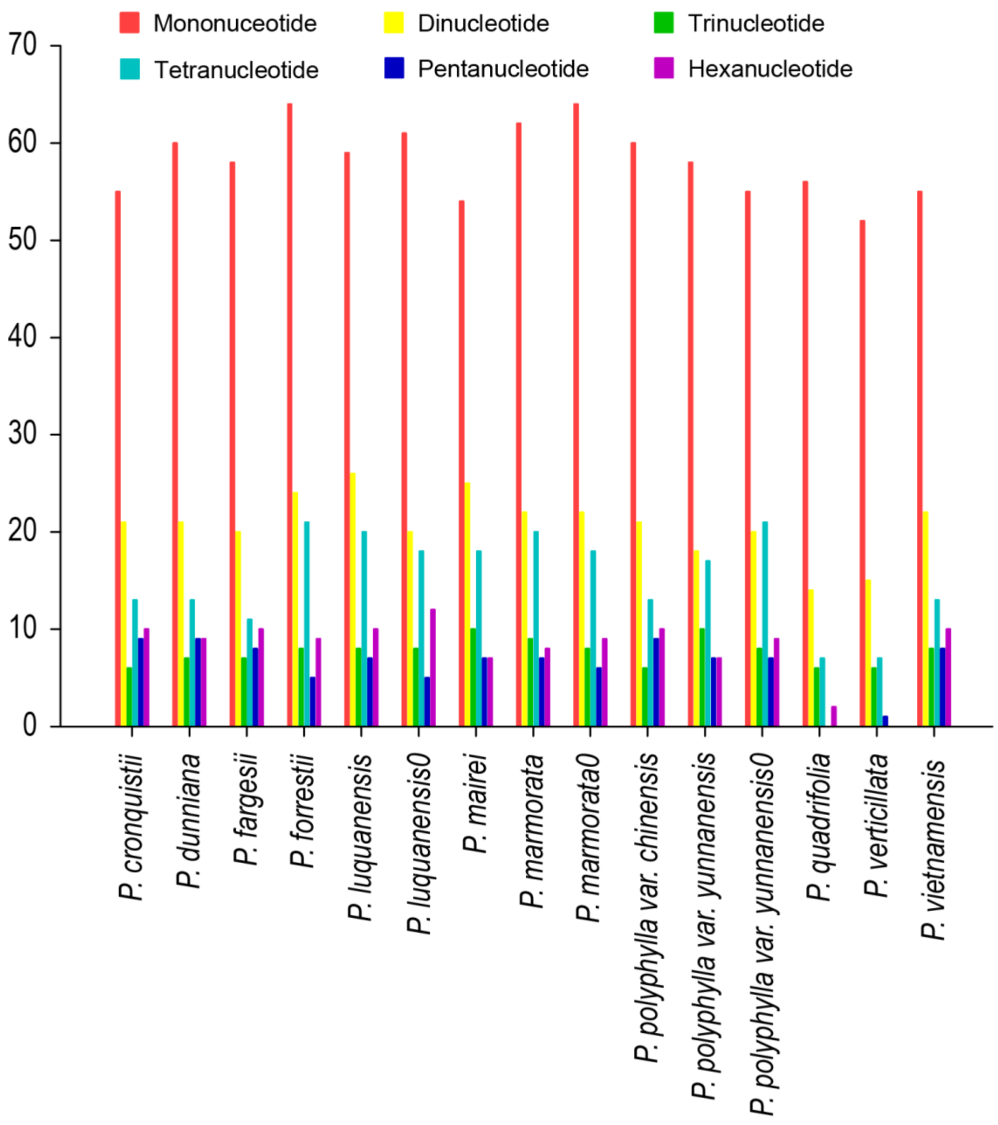

Figure S2 Six kinds of SSR motifs in fifteen *Paris* chloroplast genomes. *P. polyphylla* var. *yunnanensis0* was sequenced by Song *et al* (2015). *P. marmorata0*, *P. luquanensis0*, *P. polyphylla* var. *yunnanensis* were sequenced by Huang *et al* (2016). *P. marmorata* and *P. luquanensis* were sequenced in this study.

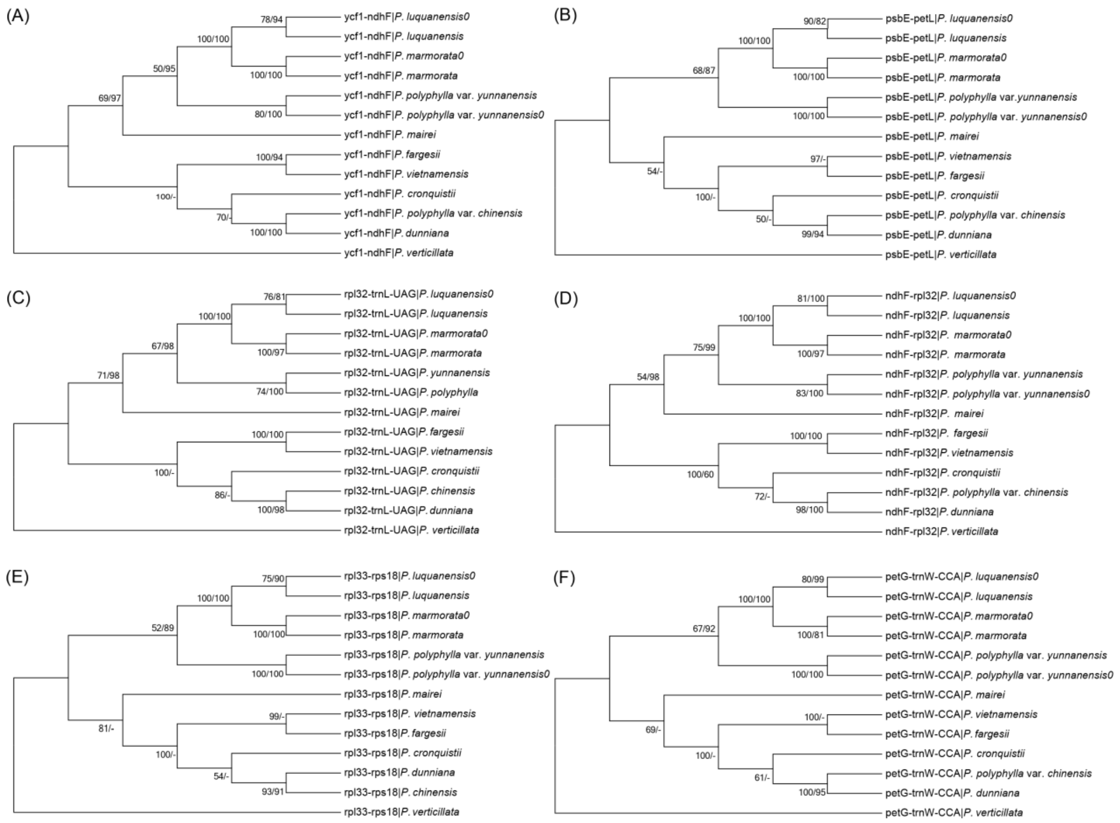

Figure S3. Phylogenetic trees of genes spacer regions harbored repeat sequence, using NJ (bootstrap values on the left of slashes) and ML (bootstrap values on the right of slashes) algorithms.

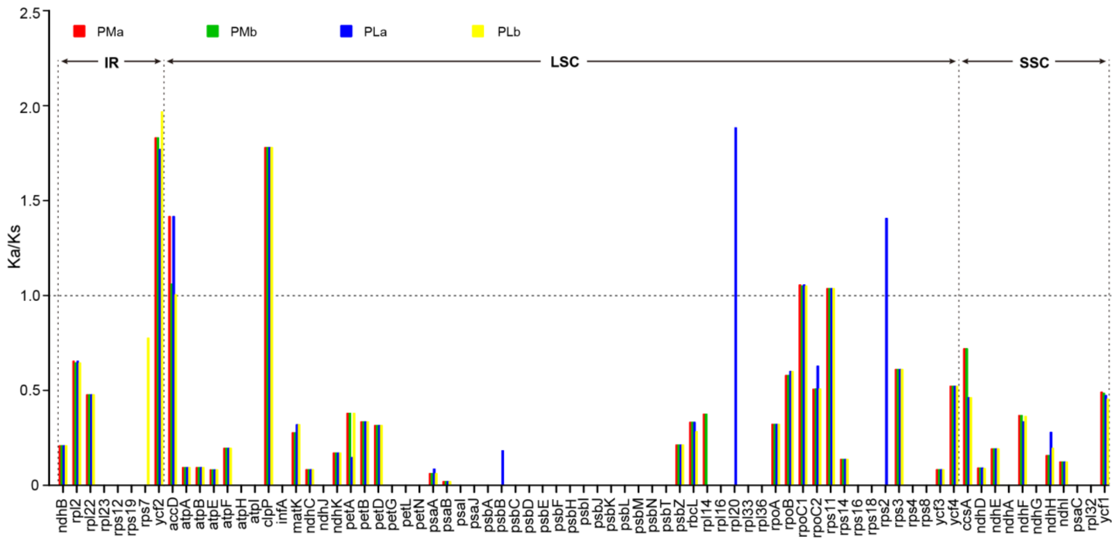

Figure S4. The K_a/K_s ratios of 71 protein-coding genes from chloroplast genome of Sect. *Marmorata*. Red bars and blue bars denote *P. marmorata* (PMa) and *P*. *luquanensis* (PLa) sequenced previously; green bars and yellow bars denote *P. marmorata* (PMb) and *P*. *luquanensis* (PLb) sequenced in this study.

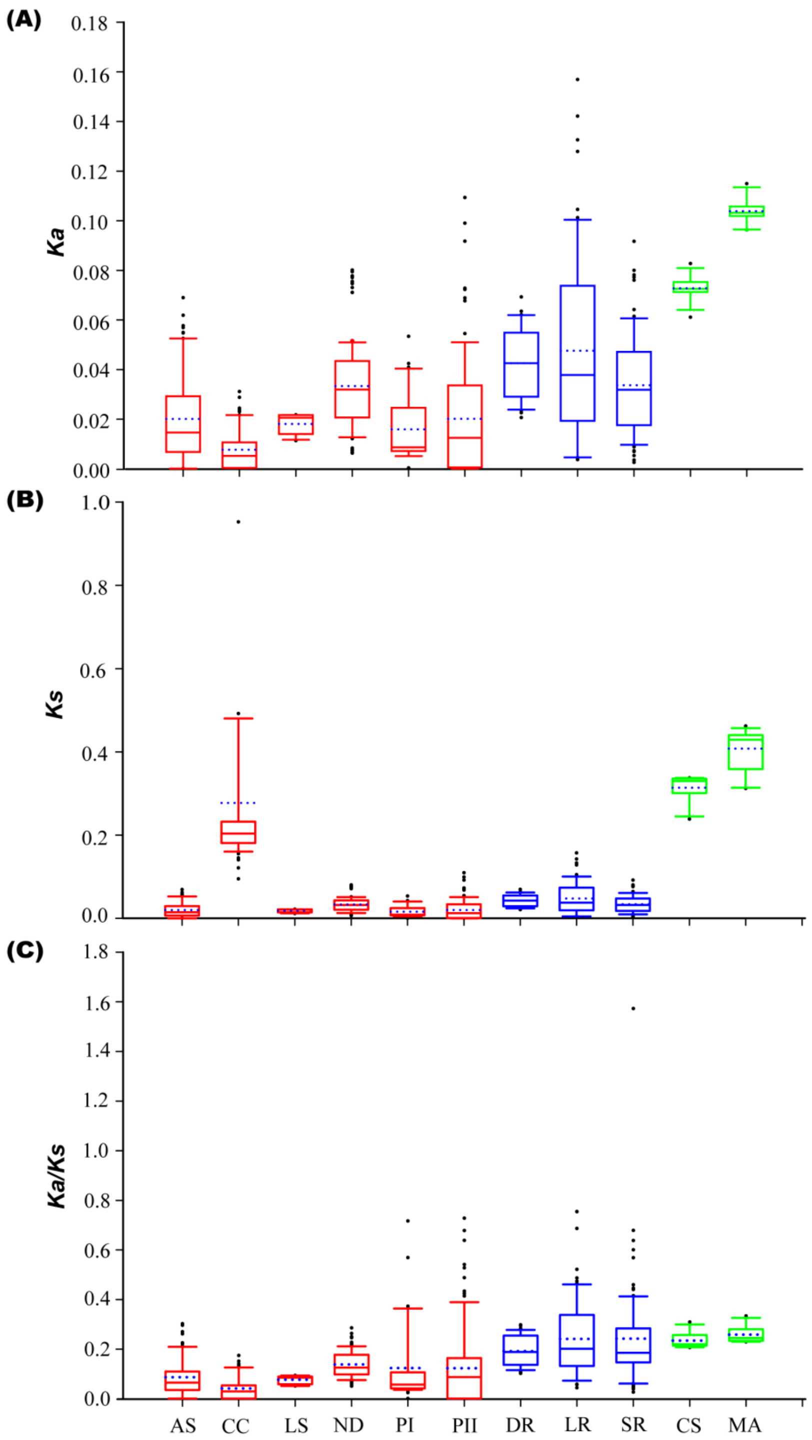

Figure S5 Comparison of K_a, K_s, and K_a/K_s ratios of Sect. *Marmorata* chloroplast genes. (A-C) denote K_a, K_s, and K_a/K_s, respectively. Red highlight boxplots indicate photosynthesis genes, green ones indicate genes involved in self-replication, and blue ones indicate other genes. SR: small subunit of ribosome, LR: large subunit of ribosome, DR: DNA-dependent RNA, TF: translational initiation factor, ND: NAPH dehydrogenase, PI: photosystem I, PII: photosystem II, CC: cytochrome b/f complex, AS: ATP synthase gene, LS: large subunit of rubisco, SA: subunit of acetyl-CoA, CS: cytochrome synthesis, CT: c-type cytochrome synthesis, PR: protease, MA: maturase, CO: conserved ORF.
